# Men, trans/masculine, and non-binary people’s experiences of pregnancy loss: an international qualitative study

**DOI:** 10.1186/s12884-020-03166-6

**Published:** 2020-08-24

**Authors:** Damien W. Riggs, Ruth Pearce, Carla A. Pfeffer, Sally Hines, Francis Ray White, Elisabetta Ruspini

**Affiliations:** 1 College of Education, Psychology and Social Work, Flinders University, GPO Box 2100, 5001 Adelaide, South Australia Australia; 2 School of Sociology and Social Policy, University of Leeds, Leeds, UK; 3 Department of Sociology, University of South Carolina, Columbia, USA; 4 Department of Sociological Studies, University of Sheffield, Sheffield, UK; 5 School of Social Sciences, Westminster University, London, UK; 6 Dipartimento di Sociologia e Ricerca Sociale, Universita’ di Milano-Bicocca, Milan, Italy

**Keywords:** Men, Trans/masculine and non-binary, Transgender, Pregnancy loss, Reproduction, Miscarriage

## Abstract

**Background:**

Growing numbers of men, trans/masculine, and non-binary people are becoming gestational parents, yet very little is known about experiences of pregnancy loss among this diverse population.

**Methods:**

The study employed a cross sectional design. Interviews were undertaken with a convenience sample of 51 trans/masculine and non-binary people who had undertaken at least one pregnancy, living in either Australia, the United States, Canada, or the European Union (including the United Kingdom). Participants were recruited by posts on Facebook and Twitter, via researcher networks, and by community members. 16 (31.2%) of the participants had experienced a pregnancy loss and are the focus of this paper. Thematic analysis was used to analyse interview responses given by these 16 participants to a specific question asking about becoming pregnant and a follow up probe question about pregnancy loss.

**Results:**

Thematic analysis of interview responses given by the 16 participants led to the development of 10 themes: (1) pregnancy losses count as children, (2) minimizing pregnancy loss, (3) accounting for causes of pregnancy loss, (4) pregnancy loss as devastating, (5) pregnancy loss as having positive meaning, (6) fears arising from a pregnancy loss, (7) experiences of hospitals enacting inclusion, (8) lack of formal support offered, (9) lack of understanding from family, and (10) importance of friends.

**Conclusions:**

The paper concludes by outlining specific recommendations for clinical practice. These include the importance of focusing on the emotions attached to pregnancy loss, the need for targeted support services for men, trans/masculine, and non-binary people who undertake a pregnancy (including for their partners), and the need for ongoing training for hospital staff so as to ensure the provision of trans-affirming medical care.

## Background

Pregnancy loss affects many people each year. Pregnancy loss encompasses miscarriage, ectopic pregnancy, and stillbirth. Miscarriage occurs in 11–22% of all pregnancies [1], and approximately 2.6 million babies across the globe are stillborn each year [2]. Research on heterosexual cisgender (i.e., non-transgender) men and pregnancy specifically suggests that such men experience grief following a pregnancy loss similar to that experienced by cisgender women [3]. Yet heterosexual cisgender men face considerable barriers to support following a pregnancy loss, due to the expectation that such men should primarily focus on supporting their female partner [4], in addition to the normative expectation that men should be stoic in the face of loss [5]. These barriers result in fewer numbers of heterosexual cisgender men seeking support following a pregnancy loss, due to the fear of stigma [6].

Although a growing body of research, summarized briefly above, provides insights as to the specific needs of heterosexual cisgender men who have experienced a pregnancy loss, little is known about how men, trans/masculine, or non-binary people who undertake a pregnancy experience a pregnancy loss. This paper uses the term “men, trans/masculine, and non-binary people” to refer to people who were coercively assigned female at birth, but who report their identity as, for example, male, man, trans, masculine, transmasculine, non-binary, genderqueer, or agender.

For men, trans/masculine, and non-binary people in receipt of testosterone, concerns have been raised about its potential impact upon a pregnancy, and specifically in terms of causing a pregnancy loss [7]. This reflects concerns raised in early research with cisgender women, which found that higher levels of testosterone may be related to an increased likelihood of experiencing a pregnancy loss [8] Later research, however, has failed to find a relationship between testosterone concentration in cisgender women and pregnancy loss [9]. Importantly, not all trans/masculine and non-binary people will choose or have access to hormonal treatments, and those who undertake a planned pregnancy are likely to have ceased hormone treatment in order to become pregnant [10]. Nonetheless, the most recent research suggests reproductive outcomes for men, trans/masculine, and non-binary people in receipt of testosterone on par with those of cisgender women [11].

Previous research on men, trans/masculine, and non-binary people and pregnancy loss is minimal, and suggests aspects of pregnancy loss unique to this population. Craven’s [12] study of 54 gender and sexuality diverse people who had experienced a pregnancy loss included four transgender or non-binary people. One of these participants, a transmasculine person, noted that despite his pregnancy loss being distressing, it was also taken as a sign that his body was working (i.e., that he was capable of achieving a pregnancy). Nonetheless, he noted that social isolation related to being transmaculine meant that the pregnancy loss was not recognized by others. In their interview study with eight transmasculine people, Ellis and colleagues [13] report that half of their participants had experienced a pregnancy loss. For these participants, there was the feeling that they had been ‘betrayed’ by their body, feeling that their bodies couldn’t do what, within a cisgenderist logic, they were expected to do (i.e., that bodies assigned female are expected to be able to reproduce). Finally, skelton [14] has written about personal experiences of miscarriage. skelton resists the idea that pregnancy loss for trans people is a ‘failure of the body’. skelton instead suggests, and similar to Craven’s participant, that achieving a pregnancy, even if it results in a pregnancy loss, shows that the body can be used to achieve a given aim (i.e., pregnancy).

Given the limited body of existing research, the objective of the present paper was to explore experiences of pregnancy loss among a sample of men, trans/masculine, and non-binary people who had undertaken a pregnancy.

## Methods

### Researcher reflexivity

We are a team of researchers who, over the past decade, have undertaken research with transgender and non-binary people and/or their family members. Our focal areas include but are not limited to family formation, parenting, intimate relationships, and healthcare. As a team we are comprised of cisgender, transgender, and non-binary people, of a diversity of genders and sexualities, one of whom has conceived and carried a pregnancy. Because none of us identify specifically as men, trans/masculine, or non-binary people who have conceived, the research under present consideration was undertaken in collaboration with an advisory board that included men, transgender and non-binary members who have conceived, and whose demographic characteristics reflect greater diversity than those represented solely among the authors of the present work.

### Study design

The broader study was a cross-sectional qualitative study of a convenience sample of 51 trans/masculine and non-binary people. Inclusion criteria were (i) identifying as trans/masculine or non-binary, (ii) having undertaken at least one pregnancy following a gender transition, (iii) living in Australia, the European Union (including the United Kingdom), the United States, or Canada, and (iv) being aged 18 years or older. Participants were recruited via posts on Facebook and Twitter, and flyers circulated both via researcher networks and by men, trans/masculine, and non-binary community members. All recruitment information was circulated in English. Recruitment continued until all participants who responded to the circulated information had been interviewed.

Participants in the U.S. and Canada were paid $25-$50 to participate; participants of color were compensated at a higher rate due to targeted recruitment aims for the study and to reflect structural constraints to participation (e.g., U.S. history of racist research exploitation and increased requests for participation) faced by participants of color. Participants in the European Union and Australia were not compensated for their participation. This reflected research norms at the institutions in these countries; compensation for participation in social research is often understood as a form of potential coercion.

### Data collection

Semi-structured interviews were undertaken either in person or remotely via Skype, Whereby or Zoom, by a research associate of the first author (for Australian interviews), by the second author (for interviews in the European Union including the United Kingdom), or by the third author (for interviews in the United States and Canada). Interviews were undertaken in English between June 2018 and October 2019. In terms of interview questions specific to the present paper, a general question was asked about experiences of undertaking a pregnancy, with specific follow up probe questions about pregnancy loss. Interviews ranged from less than sixty minutes to over three hours, with an average length of 100 min. Interviews were transcribed by a professional service, and participants either chose their own pseudonym, or were allocated a pseudonym if they did not opt to choose their own. Participants were also asked about pronouns, with most participants using either he/him or they/them. These pronouns are used in reporting the findings below.

### Analytic approach

For the purposes of the present paper, responses to the interview questions outlined above where a participant indicated that they had experienced at least one pregnancy loss were extracted for analysis. Importantly, while this question was purposively included in the interview schedule, and then purposively selected for analysis in the present paper, the analysis itself was inductive: it did not begin with a specific hypothesis to test, nor, given the limited previous research, an indicative list of likely topics that would be developed from the data. Of the total sample, 16 participants (31.2%) reported that they had experienced at least one pregnancy loss and were included in the sub-sample for the present paper.

Having extracted interview responses in relation to pregnancy and loss from the 16 participants, the first author then coded the data according to the approach to thematic analysis outlined by Braun and Clarke [15]. The first step in this process involves familiarization with the data set through repeated readings. The first author read all of the extracts three times, looking for repeated topics or codes. Through this process thematic saturation [16] was achieved following repeated reading of data extracts from 12 participants, however all 16 participant extracts were included for matters of completeness. Codes identified are included in Table 1. Having developed codes based on repeated readings of the extracts, the first author then shared these codes with the second and third authors, who confirmed the codes as representative of the data set in terms of core topics.

**Table 1:** Participant demographics

Pseudonym	Region	Gender	Sexuality	Partner	Gestational age at which pregnancy loss occurred	Number of live births
Trent	Australia	Non-binary	No response	Single	3 weeks	1
Rich	Australia	Male	Bisexual/queer	Cisgender woman	7 weeks	1
Fred	Australia	Trans man	Pansexual or bisexual	Cisgender man	8 weeks	2
Charlie	EU	Fluid / masculine leaning	Human-sexual	Transgender man	23 weeks	3
Lewis	EU	Transmasculine	Pansexual (preference for men)	Transgender man	23 weeks	1
Moddy	EU	Agender / non-binary/non-gendered	Asexual/queer	Casual partners	14 weeks and 8 weeks	4
Noam	EU	Trans man	Bisexual/pansexual	Cisgender man	13 weeks	2
Will	EU	Non-binary / trans man	Bisexual	Cisgender woman	7 weeks and 5 weeks	1
Dan	EU	Trans man	Gay	Transgender man	16 weeks	Pregnant at time of interview
Cole	US	Male	Gay	Cisgender man	6 weeks	3
Luke	US	Trans male	Gay	ex: cisgender man	10 weeks (3 additional earlier miscarriages)	2
Pete	US	Trans guy/F-to-M	Queer	Cisgender man	6 weeks and 5 weeks	2
Dee	US	Trans/ gender non-binary	Queer	None	8 weeks and 12 weeks	1
Gage	US	Trans man/transmasculine	Queer	Cisgender woman	8 weeks and 8 weeks	1
Benjamin	EU	Male/trans male		Cisgender woman	6 weeks	Pregnant at time of interview, has since given birth
Markus	EU	Man	Fluid / Pansexual / Bisexual	Cisgender man	3 weeks	Pregnant at time of interview, has since given birth

Having confirmed the codes developed, the first author then developed themes based on the codes. While codes encompass broad salient topics repeated across the data set, themes by comparison organize codes into logical and coherent sets of information. Themes developed are indicative of topics seen as salient by researchers, rather than as being exhaustive of all possible readings of the data set. Codes and themes were not mutually exclusive across participants: some participants gave interview responses that were located within more than one code or theme. For the present paper, ten key themes were identified through a process of repeated readings of the initial coded data, and a process of distilling codes into coherent thematic groupings. The ten themes are outlined in Table 1. Having identified these themes, the first author again shared them with the second and third authors, who confirmed the thematic structure. The first author then selected indicative extracts to include in the presentation of the findings below, which are accompanied by commentary on how the extracts selected demonstrate each of the themes developed, focusing on semantic as opposed to latent meaning.

## Results

### Participants

The average age (at the time of their interview) for the participants included in the sub-sample was 35 years (range 23–49). Of the participants included in the sub-sample, 10 had experienced one pregnancy loss, and 6 had experienced more than one pregnancy loss. Of the 24 pregnancy losses experienced across all 16 participants in the sub-sample, 19 were early term losses (12 weeks or less), and five were late term losses. Of the participants included in the sub-sample, 15 had experienced a live birth either prior to or following a pregnancy loss. Table 2 provides other demographic information about participants (with gender and sexuality both being participant self-descriptions).

**Table 2:** Structure of codes and themes and number of participants situated in each

Code	# of participants	Theme	# of participants
1: Participant introducing pregnancy loss as a topic	6	Pregnancy losses count as children Minimising pregnancy loss	4 2
2: Perceived factors leading to a pregnancy loss	7	Accounting for causes of pregnancy loss	7
3: Emotional responses to pregnancy loss	16	Pregnancy loss as devastating Pregnancy loss as having positive meaning Fears arising from a pregnancy loss	11 6 4
4: Formal experiences of support following pregnancy loss	7	Experiences of hospitals enacting inclusion Lack of formal support offered	4 3
5: Informal experiences of support following pregnancy loss	8	Lack of understanding from family Importance of friends, including those with lived experience	4 4

### Themes

#### Pregnancy losses count as children

For some participants, when asked about their family make-up, there was a clear orientation towards referring to pregnancy losses as indicative of children. In other words, rather than minimizing a pregnancy loss or discounting pregnancy losses by not counting them as children, some of the participants very much included pregnancy losses as part of their description of their family.

Charlie, for example, when asked about children, stated “I’ve had two that are here today, and two that aren’t. So I still count them”. Similarly, Luke stated that “I’ve given birth to two living children and we had four miscarriages and six or seven chemical pregnancies, and then my youngest, she was a twin, we lost the twin at ten weeks”. Other participants, whilst not as clearly labeling pregnancy losses as children, made comparisons that facilitated the conceptualization of pregnancy losses as equated with children, such as Moddy, who drew a comparison between their own pregnancy loss, and a relative’s experience of losing a child as a result of sudden infant death syndrome.

#### Minimising pregnancy loss

In contrast to the previous theme, a smaller number of participants appeared to minimise pregnancy losses. This is not to suggest that pregnancy losses had no meaning, but rather that pregnancy losses were not treated as children by these participants. Rather, it would seem that a pregnancy loss was situated primarily as a biological function, separate to broader accounts of family or children.

Lewis, for example, when describing a pregnancy loss, noted that “there were some issues before, like what I now sort of see as a miscarriage, but it was like it was just like a really irregular period with a massive clot”. Trent spoke about having had two ultrasounds, one where there was a heartbeat and another where there was no heartbeat, before methodically describing the process of dilation and curettage. In a similar way, Pete shared that “I carried the pregnancy for just six weeks and had a miscarriage. We understood that happens a lot, so we weren’t too worried about it”.

#### Accounting for the causes of pregnancy loss

In terms of this next theme, it is important to note that participants were not asked to explain the cause of their pregnancy loss(es). Nonetheless, almost half of the participants oriented to the topic of causes naturalistically when speaking about the experience of pregnancy loss. Accounts of potential causes largely involved conjecture on the part of participants, though some participants noted that medical professionals had given an indication of why a pregnancy loss may have occurred.

When accounting for causes, some participants normalized pregnancy loss, suggesting that it was for many people a routine and expected aspect of reproduction. An example of this was Pete, who noted that his fertility specialist suggested it was likely just due to his age, or Cole, who suggested that in the immediate months after a person ceases taking testosterone, “our bodies can get pregnant, but they are not [yet] ready to actually carry a baby to term” (though as noted earlier in this paper, even this claim may not reflect the latest research evidence [11]). Other participants provided specific examples of what they believed to be the causes of a pregnancy loss, and these typically related to stressful life events. Charlie, for example, suggested that he experienced pregnancy losses because his body wasn’t healthy and he wasn’t “eating right”. Moddy reported that they experienced a pregnancy loss after being pushed down a flight of stairs by an adult child. Dan felt that the stress of illness and a death in the family had resulted in a pregnancy loss: “I was ill three weeks prior, so we put it down to that… and then the day before [the miscarriage] I actually lost my cousin… so we put me getting stressed and everything down to rushing back”.

#### Pregnancy loss as devastating

In terms of emotional responses to experiencing a pregnancy loss, a majority of participants suggested that a pregnancy loss was devastating. Participants typically used strong emotive language when speaking about pregnancy losses, and this was particularly true for participants who did not minimize a pregnancy loss, but rather saw it as a significant life event.

Charlie stated that following his pregnancy loss, he went “off the rails, absolutely nuts”. Will described his pregnancy loss as “heartbreaking”, noting that “the first time I had a positive test… I of course was jumping the gun and making all these plans and I was pretty devastated, you know I was devastated and it was horrible”. Ben described his experience of pregnancy loss as “traumatizing”, and went on to suggest that the pregnancy loss felt like the “loss of an option” in terms of having a child. Lewis noted that “I was devastated, even though I didn’t know about it until it happened, and it must have been really early. Then I had a couple more [pregnancy losses] before [I gave birth to child], but really early [losses]. They were quite hard to deal with”.

#### Pregnancy loss as having positive meaning

By comparison, a smaller number of participants suggested that a pregnancy loss could be seen as having positive meaning. This specifically related to concerns by some of the participants that having previously been prescribed hormones might have meant they would not be able to conceive. Given the relative lack of certainty that has previously been expressed in research about the impact of testosterone on the capacity to become pregnant [7], it is not surprising that some participants had concerns that were to a degree allayed by eventually becoming pregnant, even if for some it ended in a pregnancy loss.

Will, for example, suggested that “It felt like, [the pregnancy loss] felt more positive than negative. It was sad, but more ‘like this can happen’. It felt like we had a bite, like fishing”. Markus noted that they had been aware that, given their age, conception might be difficult, leading them to suggest that “I was just thankful that it worked at all, because it was also like, oh, I didn’t know about my fertility. So it was, it was always, even if it would lead to miscarriage, it was like yeah, but at least I mean my egg and his sperm, yeah it works”. For Gage, a pregnancy loss was positive in two senses: he felt that, at the time, he and his partner “weren’t quite ready” for a child, but that the pregnancy loss really made it clear to his partner in particular that she wanted to have a child.

#### Fears arising from a pregnancy loss

For some participants, there were emotional responses that extended beyond the fact of the pregnancy loss. This typically involved more generalized fears about being a pregnant man, trans/masculine, or non-binary person. Specific to this diverse population, fears related to the possibility that pregnancy might not be a possibility, and also to the potential for the recording of information about a pregnancy loss to force disclosure to other people that a participant was transgender.

Trent, for example, was concerned about having a medical record in relation to pregnancy loss, and whether or not that would serve as a marker of his gender history. Similarly, when he was experiencing a pregnancy loss, Pete noted that going to the hospital “was a big decision, considering I was going to have to out myself to a hospital full of strangers. I have never had to go to the emergency room as a trans guy before and it’s always been a huge fear of mine”. Both Markus and Dan noted that in subsequent pregnancies, and especially in the first trimester, that they were particularly worried that they would experience another pregnancy loss. Markus, for example, noted that “The first trimester was kind of difficult for me because one thing was the two miscarriages I had before, so I couldn’t stay calm or whatever. I was always worried a bit”.

#### Experiences of hospitals enacting inclusion

Less than half of the participants spoke about receiving formal support in relation to pregnancy loss. Interestingly, of those participants in the present study who did receive formal support from a hospital following a pregnancy loss, a majority spoke about having positive experiences with hospitals. Such experiences typically involved hospital staff acting in trans-affirming ways, and providing reassurance that the pregnancy loss was not a trans-specific phenomenon.

Noam, for example, shared that the hospital he attended took extra care of him, including that when they were writing up his discharge papers, came to ask specifically about his pronouns. Cole noted that hospital nursing staff had been especially supportive, specifically telling him “’This is super common. One in four pregnancies, especially this early on, do not end up being viable. You did nothing wrong. This is not because you’re trans. There’s no reason why you should believe that this would happen’. I mean, just like they were really, really, really lovely”. Pete spoke about a nurse who refused to accept that the hospital computer had him marked as female, and went to lengths to try and amend this in the system: “The code in the computer for miscarriage would only populate to a female patient. [The staff member doing intake] was like ‘I can’t do this to a female patient. You’re a male patient’… So she called IT and had IT come up to try and override the systems, to get me the right diagnostic code”.

#### Lack of formal support offered

A smaller number of participants, however, noted that they received little or no support from hospital staff. This echoes research with both cisgender men and women [3, 17], who have reported that support following a pregnancy loss is often less than forthcoming. In terms of a lack of support offered by hospitals, this was particularly acute when it came to ongoing support post release from hospital, with participants noting that they desired, and indeed needed, ongoing support, support that was not offered. Moreover, when offered support did not appear mindful of the specific needs of men, trans/masculine, and non-binary people in regard to pregnancy loss, including the partner of the person who was pregnant.

Moddy, for example, stated that “There was no support for miscarriages at that time. I got sent away. There was no offer of counseling, no ‘do you need to talk about it’, none of that. It felt very, very closed off”. Jay stated that they “didn’t feel like there was anything available”, and they further noted that “I feel like what does exist is around supports for the person who was pregnant and there’s very little support for their other person, if there is another person. So I think there needs to be places for trans guys, but there also needs to be places for folks who are partnered to the person who was pregnant”.

#### Lack of understanding from family

Half of the participants commented about support (or the lack thereof it) from their family. Given that many participants felt that they did not receive adequate or any support from hospital staff, and that participants identified that ongoing support was often non-existent, a lack of support from families often compounded the sense of loss following a pregnancy loss. Given the broader social context, in which pregnancies by men, trans/masculine, and non-binary people are often marginalized or not recognized, the lack of support from families may also serve to further compound this marginalization.

Some participants spoke about unsupportive family members, and particularly mothers. Jay, for example, shared that their mother had told them that they would struggle to conceive, and when they did conceive, she told them “then you’ll probably miscarry”. This meant that when they did indeed miscarry, Jay struggled to turn to their mother for support. Similarly, Pete noted that he and his partner had decided not to tell their family “because we knew or suspected that my family was not gonna take [being pregnant] well”. When Pete experienced a pregnancy loss, he “ended up telling mom and one of my sisters… just because I was upset about it… I think because it was a kind of sad situation they were more supportive than if I had currently been pregnant… So I did have some support through them, but it was limited. I knew they weren’t crazy over the idea of me carrying, but they weren’t vocalizing it as much”.

#### Importance of friends, including those with lived experience

For other participants, however, informal support from friends was received, and experienced as vital. Given the lack of support from family that many participants experienced, support from friends was experienced as essential to coping with, and coming to an understanding of, a pregnancy loss. This was especially true when support was provided by friends who had themselves experienced a pregnancy loss, and who acknowledge it as highly emotive, rather than simply dismissing or minimizing the pregnancy loss.

Charlie, for example, noted that “Luckily I had at least two of my friends that were supporting me… It was everything that they were there… and if I didn’t have them I think I would have gone more nuts than I did”. Gage noted that his partner was “amazingly present” and that caring friends who provided “support made a huge difference”, including “one of whom had really struggled to get pregnant herself... showed up when I got home without an agenda and just said ‘Hey, I’m just gonna sit here next to you this evening and you can ignore me or not’”. Jay emphasized the benefits of telling friends, as it meant they could help foster a supportive network, and particularly people who had themselves experienced a pregnancy loss. As they noted, “the outpouring of other people was super helpful. It felt like just about anyone I’d known who tried to get pregnant… said ‘Oh yes, this happened to me as well’”.

## Discussion

The findings reported in this paper provide some support for previous research, both in terms of previous research on cisgender heterosexual men and pregnancy loss, and in terms of the limited literature on gestational parents who are men, trans/masculine, or non-binary. In terms of the former, participants in the present study commonly reported strong emotional responses to a pregnancy loss, and like cisgender men, similar to those experienced by cisgender women [3]. Different to previous research on cisgender men, however, participants in the present study did not report experiencing the expectation that they be stoic in the face of a pregnancy loss [5], nor did this appear to lead to fewer participants desiring to access support. This difference may be due to the fact that the participants undertook a gestational role, and hence the expectations placed upon them may have been more similar to those placed upon cisgender women, as opposed to cisgender men. Either way, the lack of supports noted by participants, and the importance of friends as a result, is echoed in research with both cisgender women and men [3, 17].

In terms of the limited literature on gestational parents who are men, trans/masculine, or non-binary, and similar to research by Craven [12], participants in the present study spoke about pregnancy loss as distressing, but also as a sign that their bodies were working. Conversely, the findings reported in this paper did not indicate support for previous research by Ellis and colleagues [13], which found that participants felt ‘betrayed’ by their body. In the present study none of the 16 participants who had experienced a pregnancy loss drew upon this narrative, suggesting that skelton’s [14] resistance to ‘failed body’ narratives warrants closer attention. Specifically, skelton’s [14] argument, and the findings presented in this paper, must be situated alongside ongoing resistance within transgender communities to the predominance of ‘wrong body’ narratives used to describe transgender people’s lives in media and healthcare contexts. These narratives position transgender people as having been ‘born in the wrong body’ [18]. ‘Wrong body’ narratives have been suggested to contribute to the pathologisation of transgender people’s lives, as well as framing transgender people’s bodies as a problem to be solved [18]. In the context of pregnancy loss for men, trans/masculine, and non-binary people and the logic of cisgenderism, ‘wrong body’ narratives may contribute to the sense that for some people, as Ellis and colleagues [13] found, the body has doubly failed. By contrast, in the present paper, the absence of this type of narrative would suggest potential resistance to ‘wrong body’ narratives, with pregnancy losses experienced by men, trans/masculine, and non-binary people instead framed as involving bodies that still have the capacity to achieve a certain end (i.e., the birth of a child).

These points about ‘wrong body’ and ‘betrayed’ or ‘failed’ bodies have implications for clinical practice. Specifically, the findings presented in this paper suggest that whilst a pregnancy loss can be deeply distressing for many men, trans/masculine, and non-binary people, it can also be a sign that the body is working. Clinicians will best meet the needs of trans/masculine and non-binary people who have experienced a pregnancy loss by focusing on the emotions attached both to the loss and to the possible desire to attempt another pregnancy, rather than focusing on pregnancy loss as a means to infer that trans/masculine, non-binary and men’s bodies should not be pregnant. The findings reported in this paper also suggest that clinicians will best meet the needs of men, trans/masculine, and non-binary people by acknowledging that, at least for some people, a pregnancy loss may be experienced as a child lost, signaling the need for supportive counseling for both gestational parents and their partners. Such counseling may support men, trans/masculine, and non-binary people and their partners to access further support through informal channels (i.e., friends with lived experience), as well as addressing potential barriers to support in regard to family. This may include involving family members in counseling so as to facilitate their understanding of the needs and experiences of men, trans/masculine, and non-binary people following a pregnancy loss.

These implications for practice highlight that whilst there may be some similarities to other people’s experiences of pregnancy loss (i.e., feelings of distress, mixed experiences in terms of support, differing accounts of causes), trans/masculine, non-binary, and men’s experiences as highlighted in this paper demonstrate unique points of difference. These include the importance of inclusive healthcare (i.e., asking about pronouns, refusing to accept misgendering within healthcare systems), the specific meanings that trans/masculine and non-binary people may bring to the experience of pregnancy loss (i.e., in regards to concerns about testosterone and pregnancy), and the ways in which marginalisation may negatively impact on available support (i.e., in terms of unsupportive family members).

In terms of limitations, participants experienced pregnancy losses at a diversity of gestational ages. Future research will benefit from examining more closely whether gestational age differentially impacts upon experiences of grief in terms of pregnancy loss for men, trans/masculine, and non-binary people, though research on cisgender men’s experiences suggests that gestational age is not a determining factor in terms of grief following a pregnancy loss [3]. Further, and given that age has been found to be a factor in pregnancy loss for cisgender women [19], it is important to be mindful that age may have played a role in the occurrence of pregnancy loss amongst the sample, though likely did not significantly shape *experiences* of pregnancy loss. Additionally, it is important to note that the interviews did not uniformly enquire about the relationship between conception, pregnancy loss, and receipt of hormones. Whilst research suggests that testosterone levels are unrelated to pregnancy loss for cisgender women [9], this is a topic worthy of further investigation in terms of trans/masculine and non-binary people. In contrast to these potential limitations, we would note that web-based sampling, as utilised in the study reported in this paper, is increasingly regarded as a primary and effective sampling strategy for accessing difficult-to-reach or ‘hidden’ populations to discuss sensitive issues (such as, for example, pregnancy loss). This is especially the case for members of transgender communities, who have long used the internet to build community, knowledge, and support in the context of cisgenderism.

## Conclusions

In conclusion, this paper makes an important contribution to the literature through its focused and detailed examination of trans/masculine, non-binary, and men’s experiences of pregnancy loss. The findings suggest that ongoing attention to trans/masculine, non-binary, and men’s experiences and needs in terms of pregnancy loss is warranted. This includes in terms of the clinical encounter directly following pregnancy loss, ongoing support (both formal and informal), and the importance of providing and creating spaces for and with men, trans/masculine, and non-binary people for their feelings, both positive and negative, to be explored, in order to ensure that experiences of pregnancy loss are acknowledged and heard.

Specifically, and in order to ensure a trans-affirming space for men, trans/masculine, and non-binary people, both hospital staff and staff providing grief counselling specific to pregnancy loss will benefit from training specific to working with this population. This should include a focus on the importance of asking about pronouns, advocating for system change in terms of ensuring that names, pronouns and gender can be correctly recorded, and ensuring that medical experiences following a pregnancy loss do not further compound the potential grief experienced by men, trans/masculine, and non-binary people and their partners.

## Data Availability

The datasets generated during the current study are not publicly available due to ethical concerns. Participants did not consent to having their anonymised transcripts made available to others and privacy may be compromised due to the qualitative nature of the study.
